# Supplementary material for: Protein phosphatase 5 regulates titin phosphorylation and function at a sarcomere-associated mechanosensor complex in cardiomyocytes
Source: Nat Commun. 2018 Jan 17;9:262. doi: 10.1038/s41467-017-02483-3 (PMC5772059; doi:10.1038/s41467-017-02483-3)
Supplement: Supplementary file 1 — Supplementary information [file 41467_2017_2483_MOESM1_ESM.pdf]

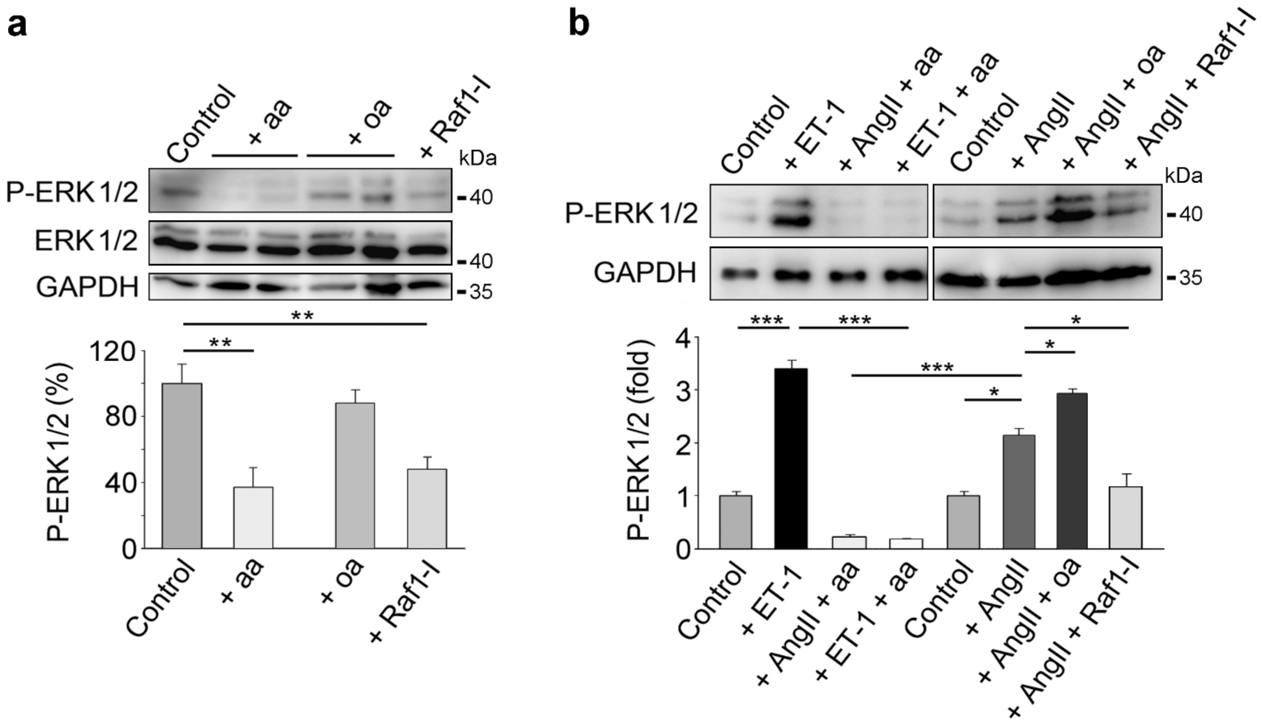

Supplementary Figure 1. Pharmacological modulation of MAPK/ERK pathway activation in neonatal rat ventricular myocyte cultures. (**a**) Expression of phosphorylated (activated) ERK1/2 and total ERK1/2 by Western blot under baseline conditions (control) and following treatment with arachidonic acid (aa; 200 µM, 2 h), okadaic acid (oa, 10 nM; 1 h), or inhibitor of Raf1 (Raf1-I, 20 µM; 1 h). P-ERK signals normalized to ERK signals (and indexed to GAPDH). (**b**) Phospho-ERK1/2 expression before and after treatment with endothelin-1 (ET-1, 10 nM; 24 h) or angiotensin-II (AngII, 1 µM; 24 h), and additional treatment with aa (200 µM; 2 h), oa (10 nM; 1 h), or Raf1-I (20 µM; 1 h). Bar graphs in (a) and (b) show phospho-ERK1/2 over ERK1/2 (indexed to GAPDH), relative to the mean expression level in control cells. Data are mean±SEM, n=3 different cell culture batches per condition; duplicate analysis per batch; *p<0.05, **p<0.01, and ***p<0.001, by Holm-Sidak method following ANOVA.

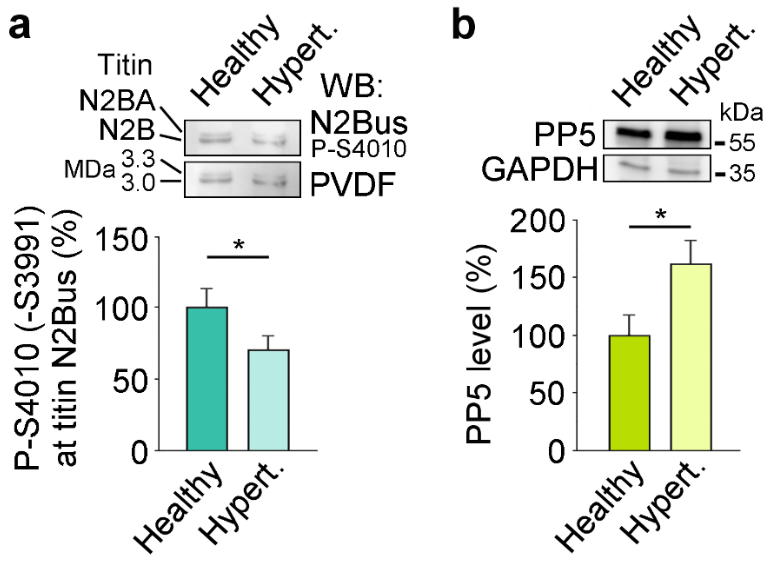

Supplementary Figure 2. Expression of phospho-titin and PP5 in healthy and diseased dog left ventricular tissue. (**a**) Site-specific N2Bus phosphorylation by Western blot in myocardial tissue from healthy canine hearts vs. hypertensive (Hypert.) hearts with diastolic dysfunction. Detection with phospho-specific antibody to human P-S4010. Bar graph shows the mean±SEM (n=5/group) phosphorylation level in hypertensive hearts relative to that in control hearts. (**b**) PP5 expression in healthy canine hearts vs. hypertensive hearts (n=5/group). Bar graph shows the mean±SEM (n=5/group) expression level in hypertensive hearts relative to that in control hearts. *$p<0.05$, by two-tailed Student's t-test.

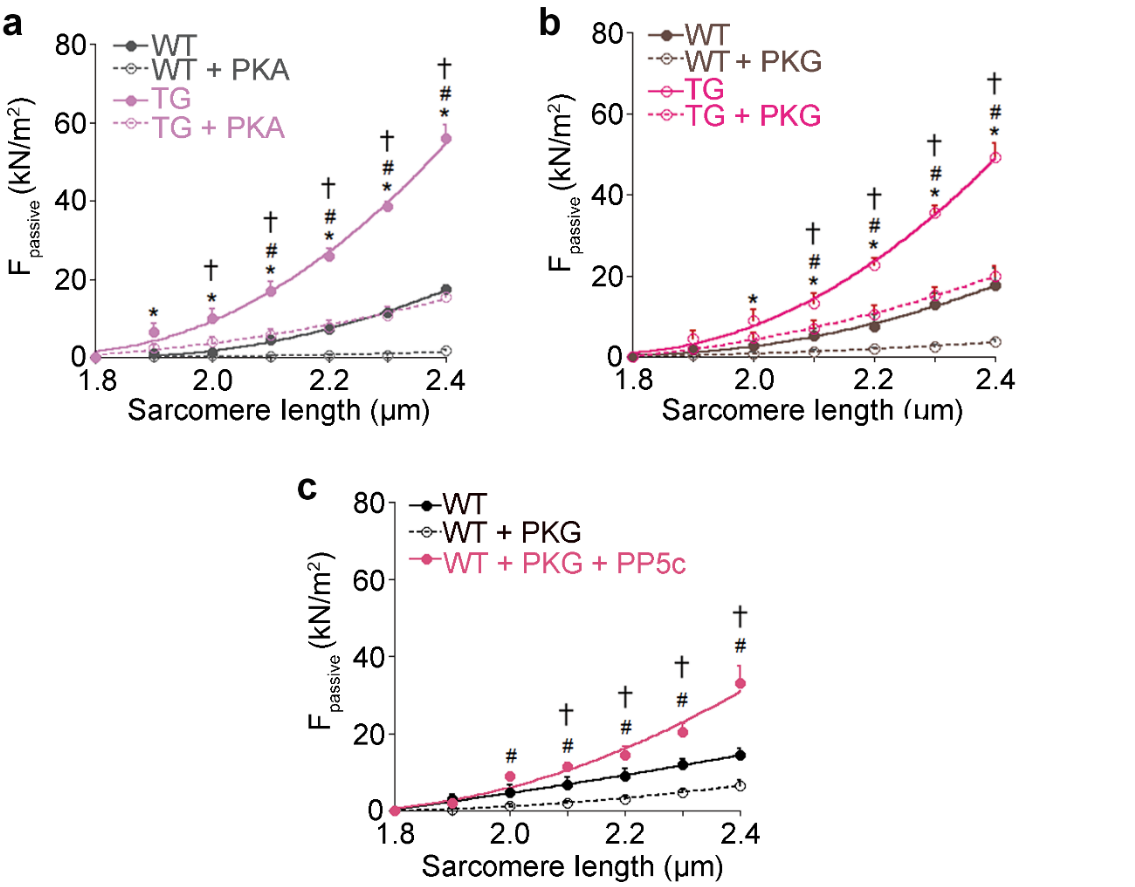

Supplementary Figure 3. Effect of PKA, PKG, and PP5c on passive tension of single permeabilized cardiomyocytes from WT and PP5 TG mouse hearts. (**a**) Passive tension ($F_{passive}$) vs. SL curves of PP5 TG and WT cardiomyocytes in relaxing solution, before and after treatment with catalytic subunit of PKA. (**b**) $F_{passive}$-SL curves of PP5 TG and WT cardiomyocytes before and after treatment with cGMP-activated PKG. (**c**) $F_{passive}$-SL curves of WT cardiomyocytes before and after treatment with cGMP-activated PKG and additional exposure to PP5c. Data are mean±SEM, n=6 cells/condition (a and b), n=5 cells/condition (c). Curves are second-order polynomial fits to the means. *$p<0.05$, TG vs. WT; $^{\#}p<0.05$, WT+PKA vs. WT in (a) and WT+PKG vs. WT in (b) and (c); $^{\dagger}p<0.05$, TG+PKA vs. TG in (a), TG+PKG vs. TG in (b), and WT+PKG+PP5c vs. WT+PKG in (c); all by two-tailed Student's t-test.

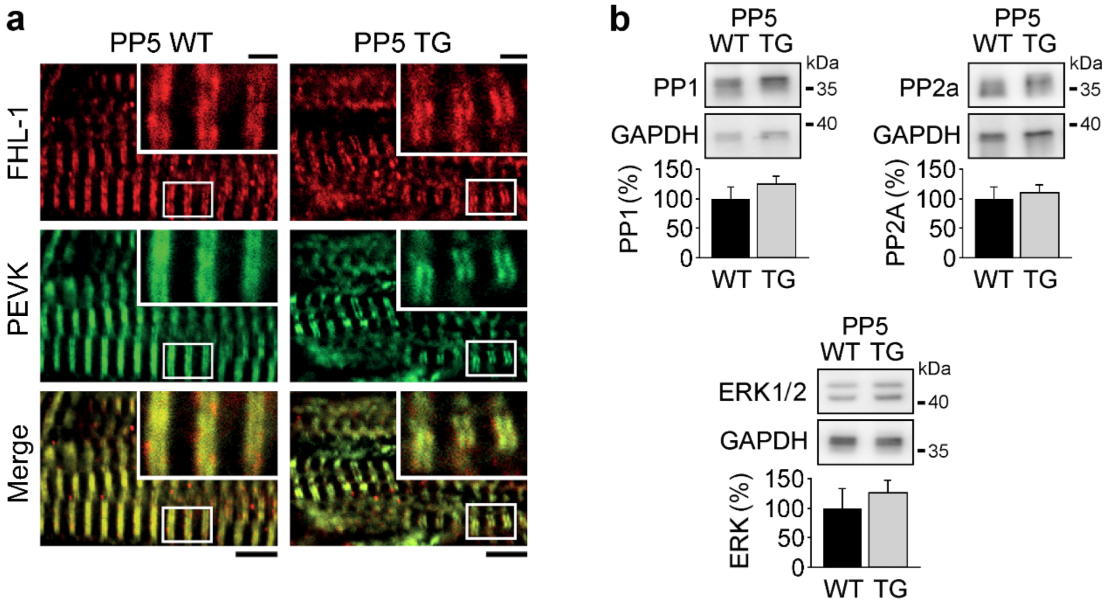

Supplementary Figure 4. Properties of signaling molecules in PP5 TG and WT mouse cardiomyocytes. (**a**) Localization of FHL-1 in cardiomyocytes from PP5 TG and WT hearts by indirect immunofluorescence. Anti-FHL-1 antibody (secondary antibody: Cy3-conjugated IgG), counterstained with anti-PEVK (titin) antibody (secondary antibody: FITC-conjugated IgG). Bars, 5 µm (main) and 1 µm (insets). (**b**) Expression levels of PP1, PP2a, and ERK1/2 in PP5 TG mouse hearts, relative to those in WT hearts, by Western blot. Bar graphs show mean±SEM, n=4 hearts/group; duplicate analysis/heart.

Figure 1b

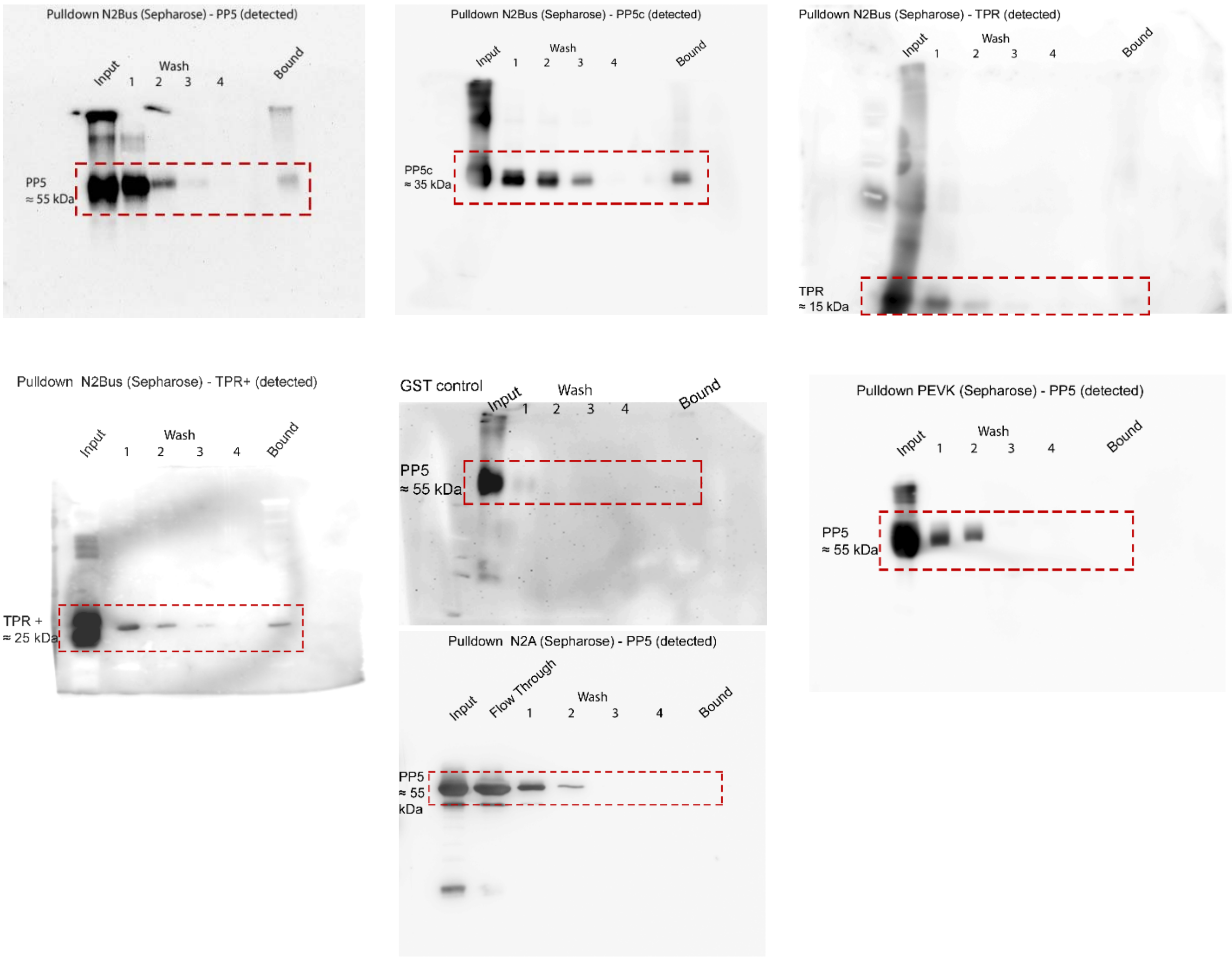

Figure 1c

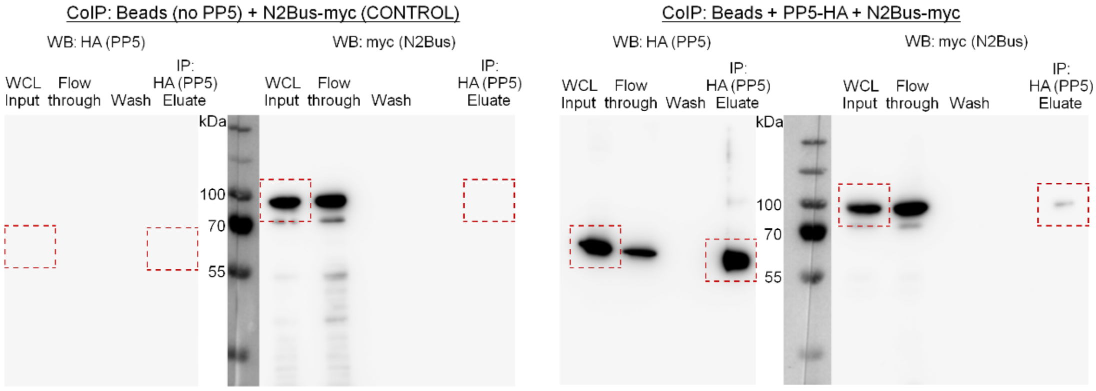

Figure 1e

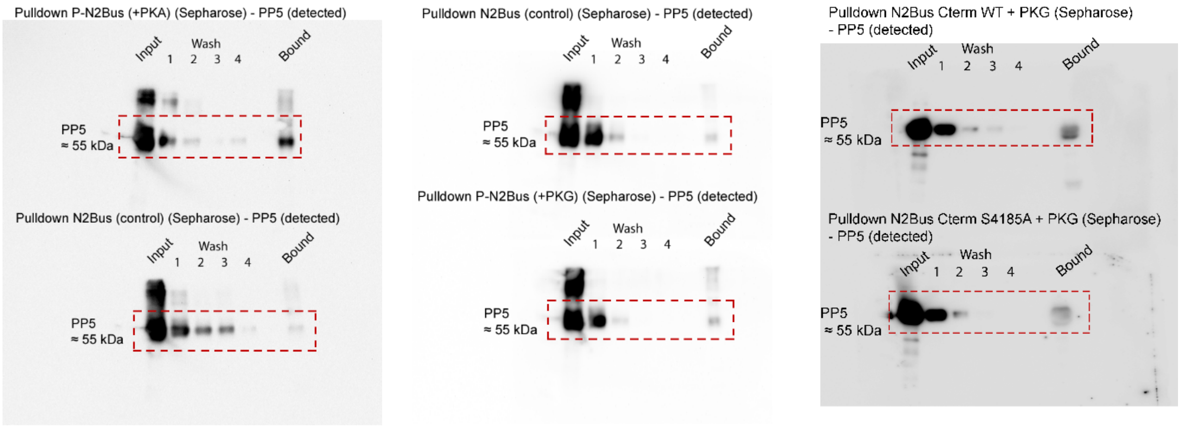

Figure 2b

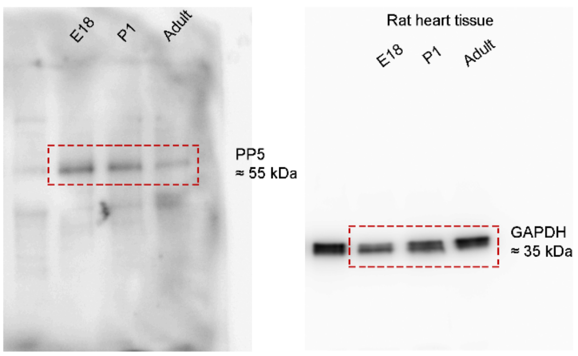

Figure 2b

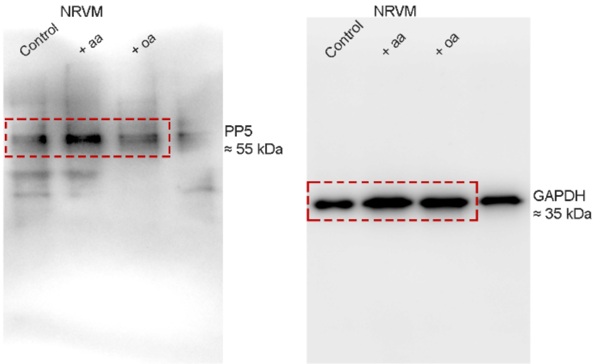

Figure 2e

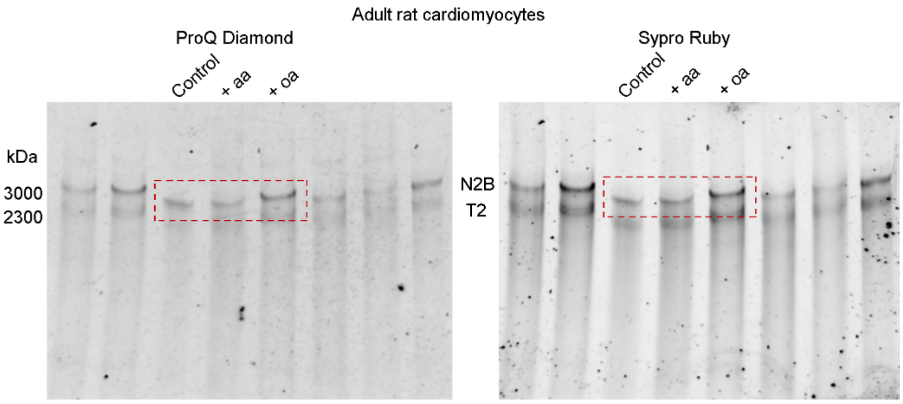

Figure 3a

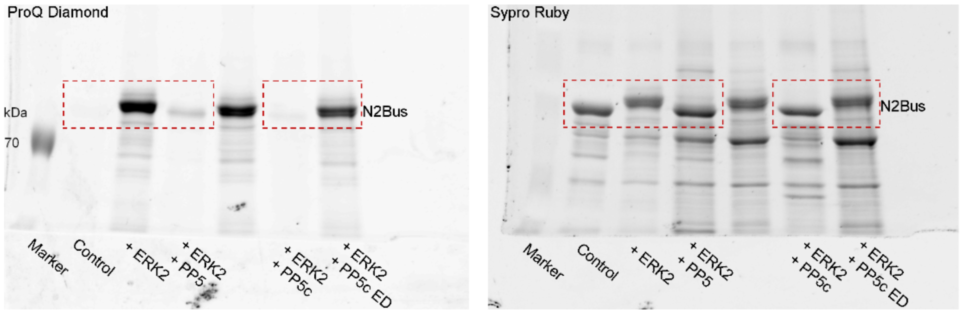

Figure 3b

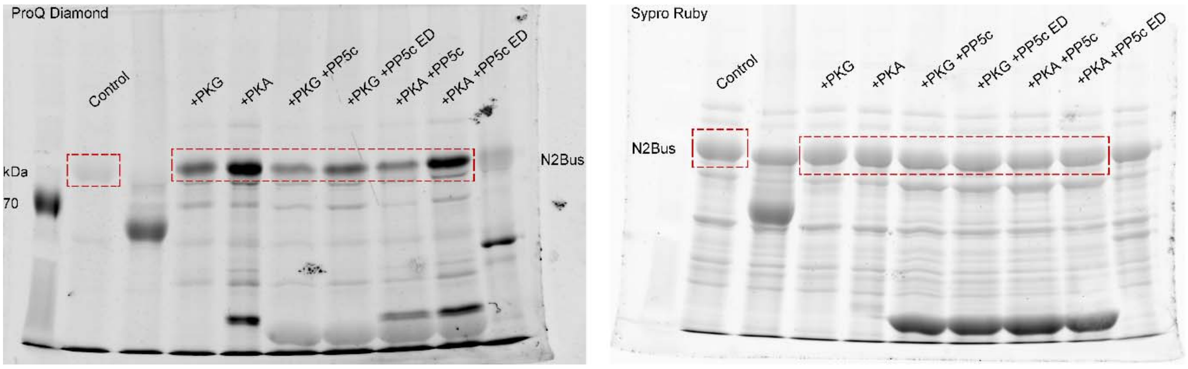

Figure 3c

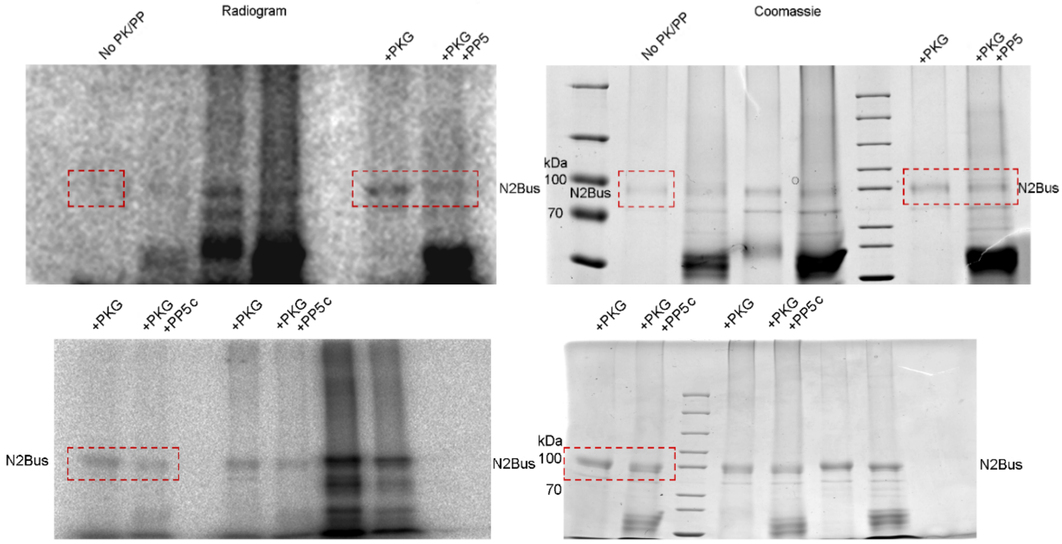

Figure 3d

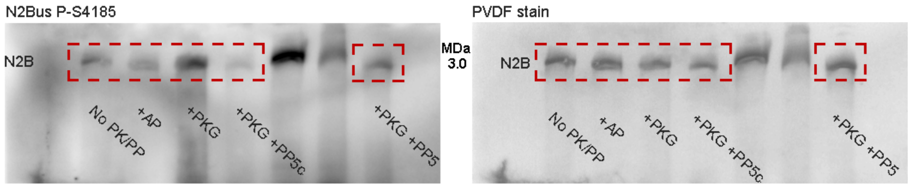

Figure 3e

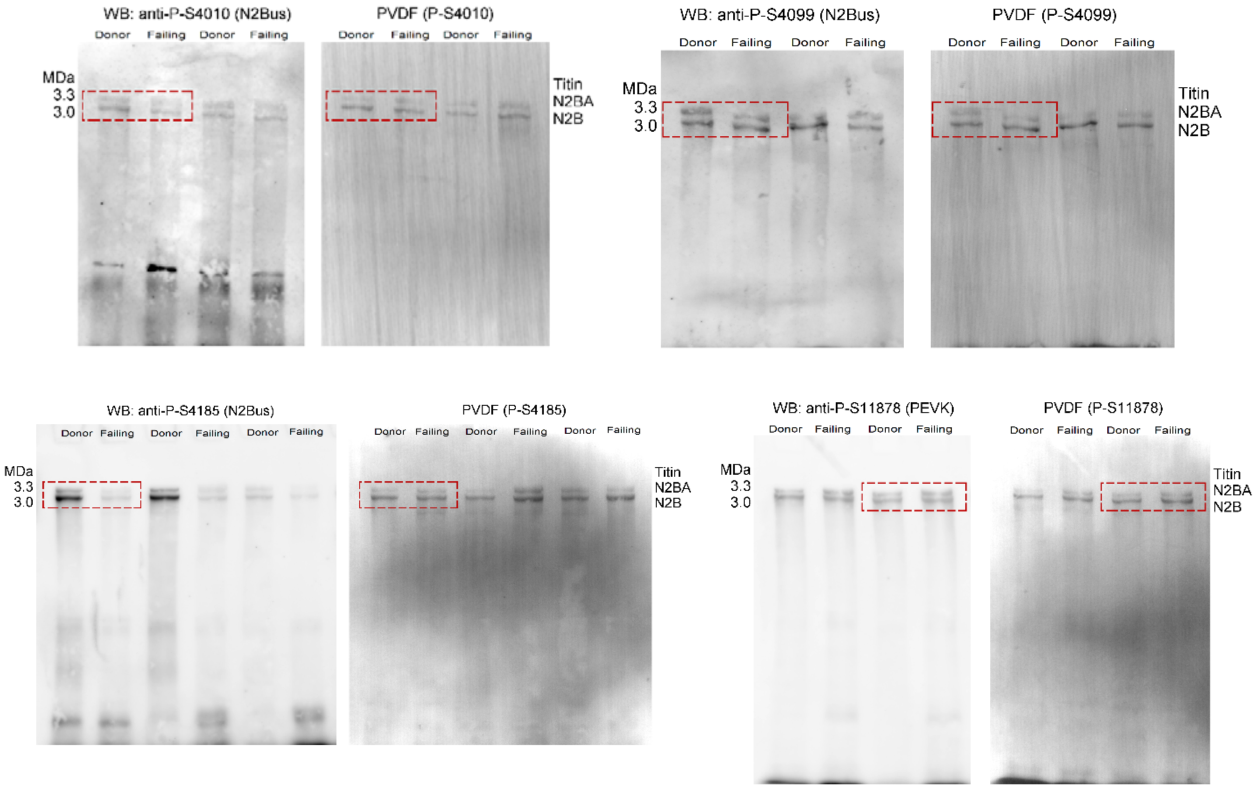

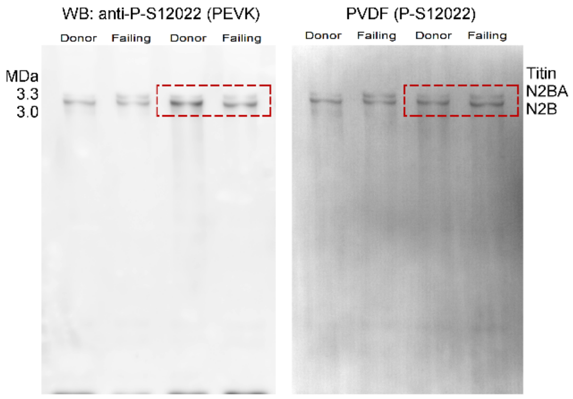

Figure 3f

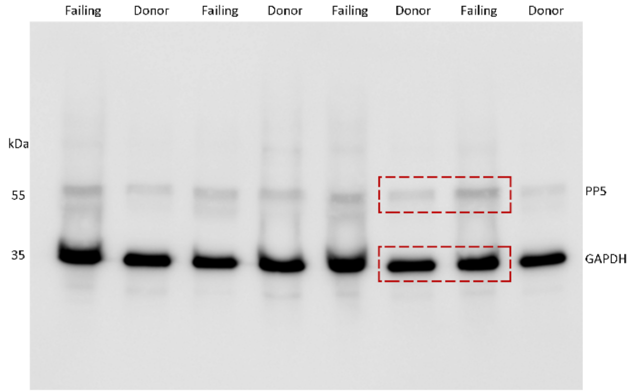

Figure 4a

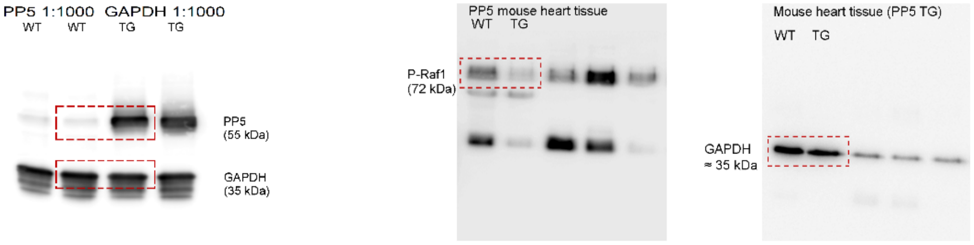

Figure 4d

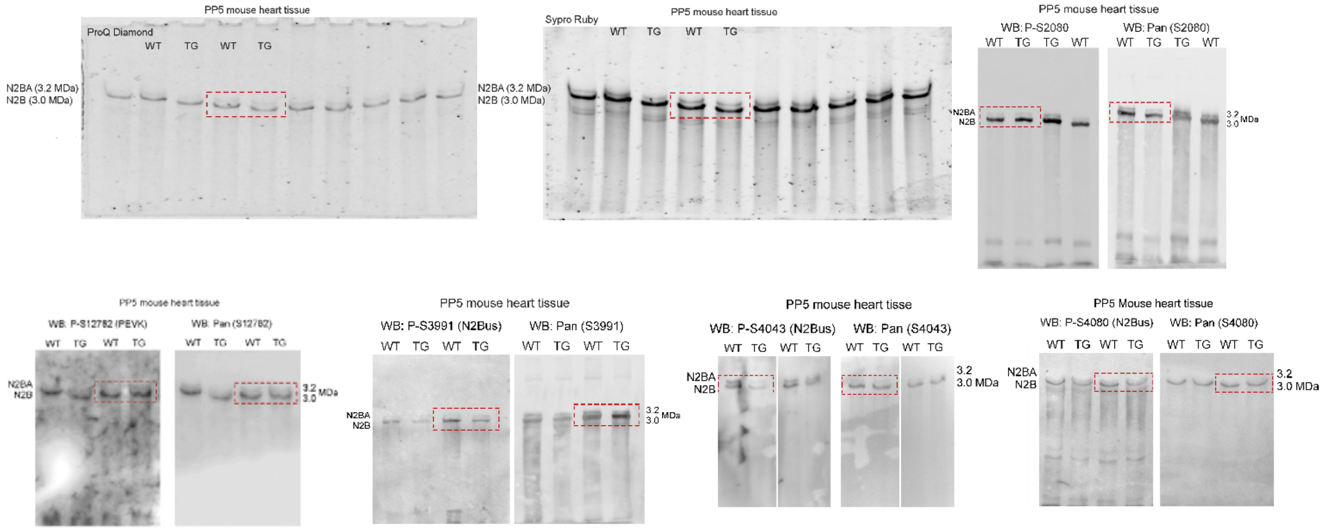

Figure 6a

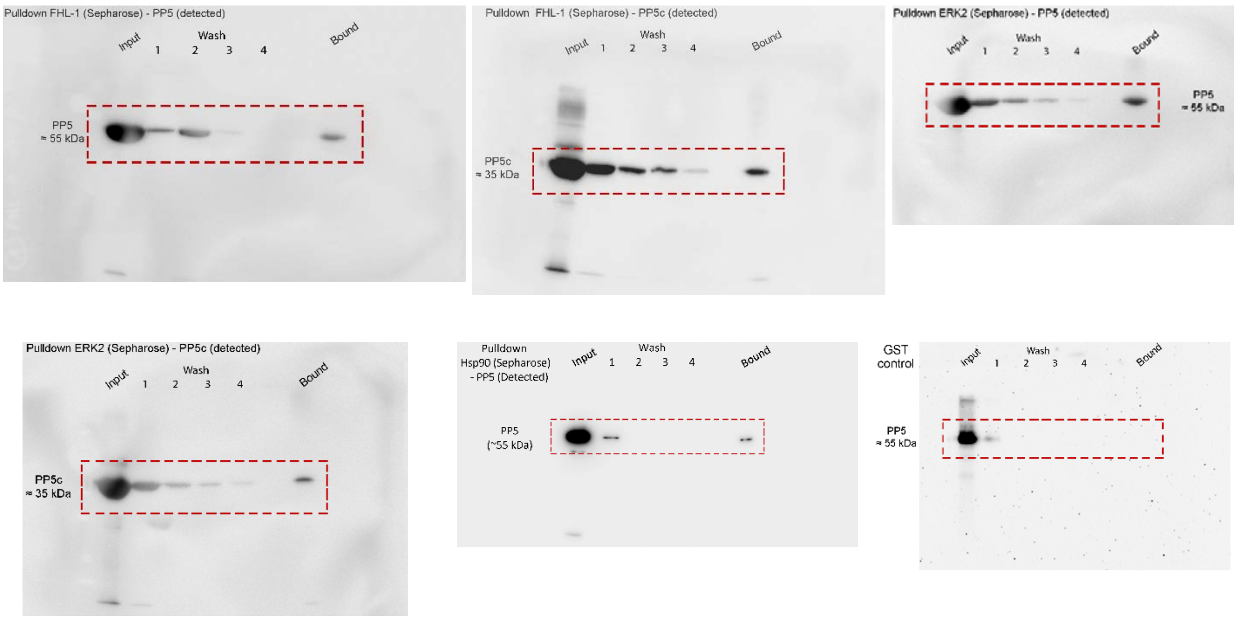

Figure 6b

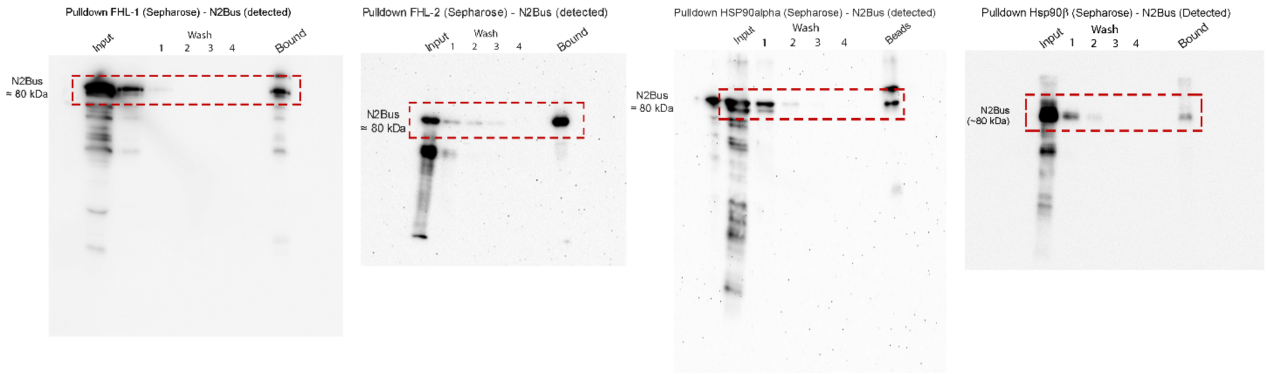

Figure 6c

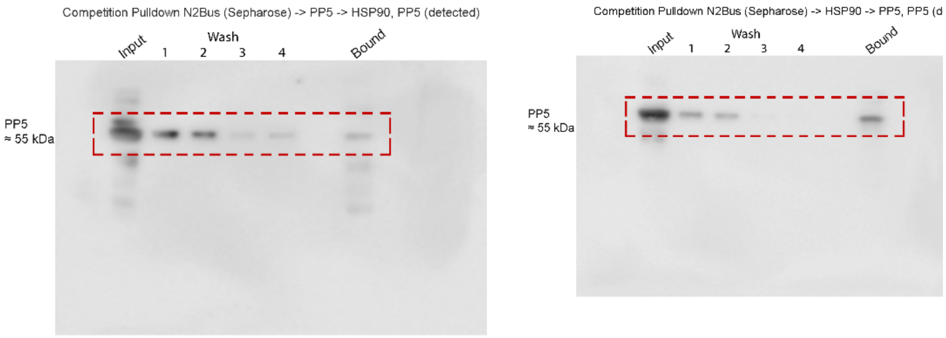

Figure 7a

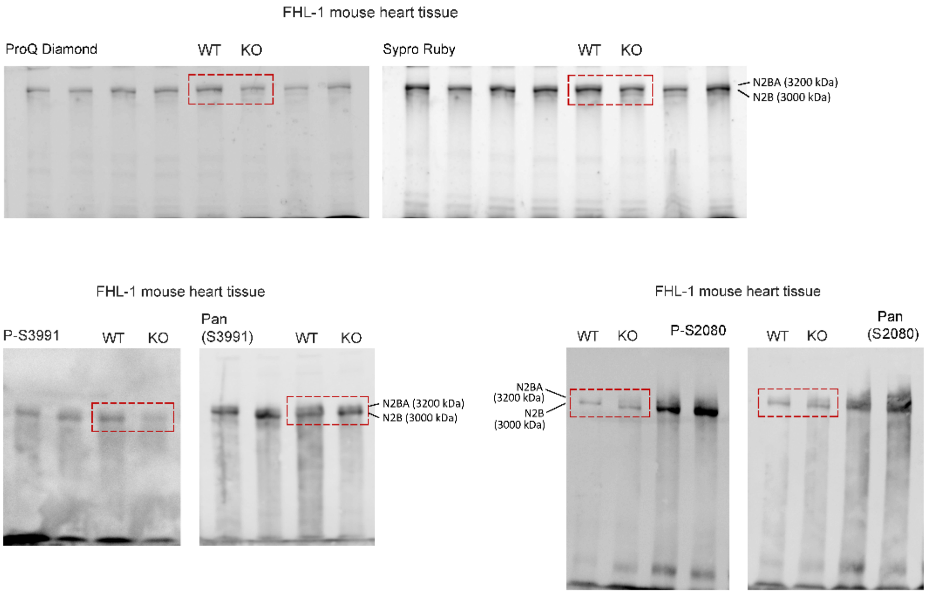

Supplementary Figure 5. Uncropped images of blots and gels used in main figures. The number of the corresponding figure is shown above the panels. Regions used for figures are boxed. Position of molecular mass marker is shown on the side (kDa or MDa).

Supplementary Figure 1a

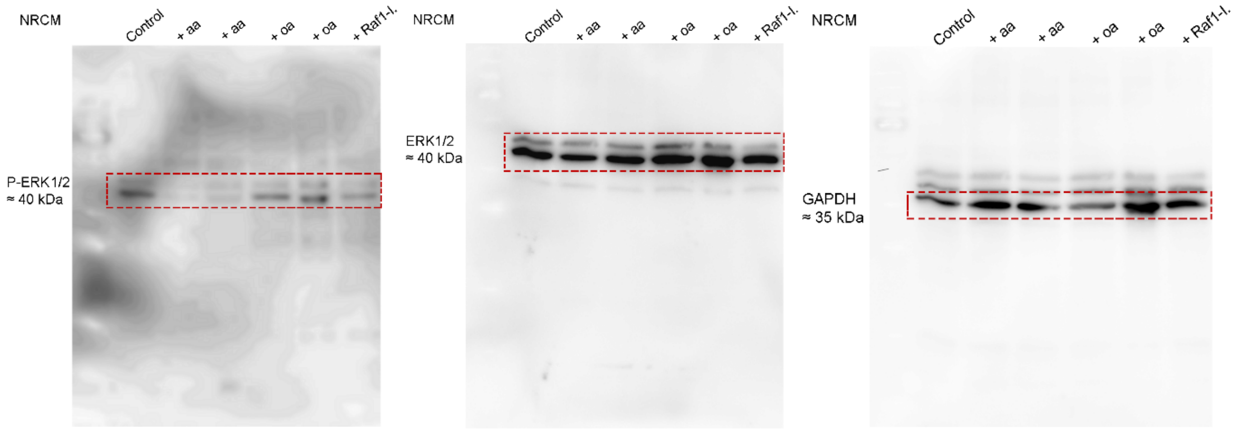

Supplementary Figure 1b

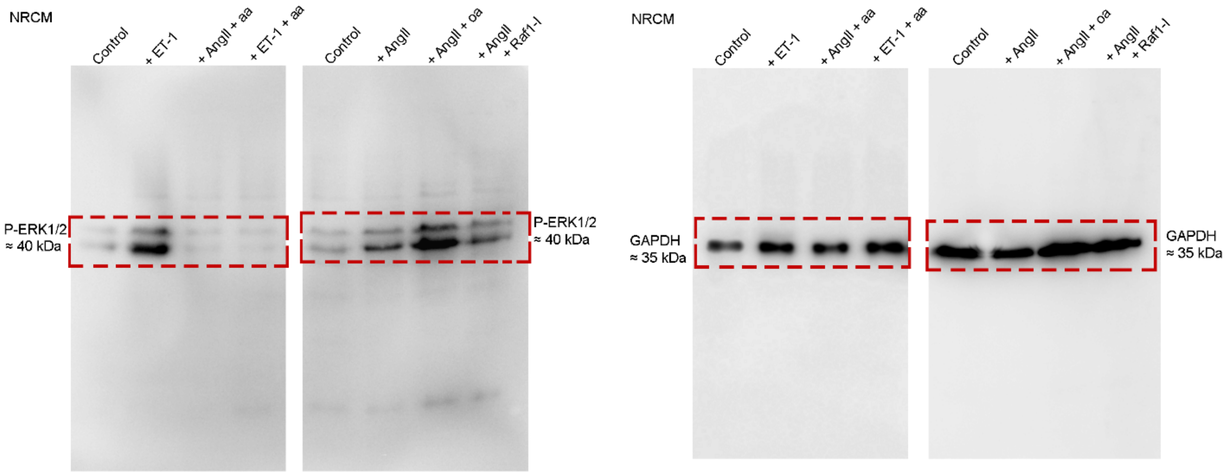

Supplementary Figure 2a

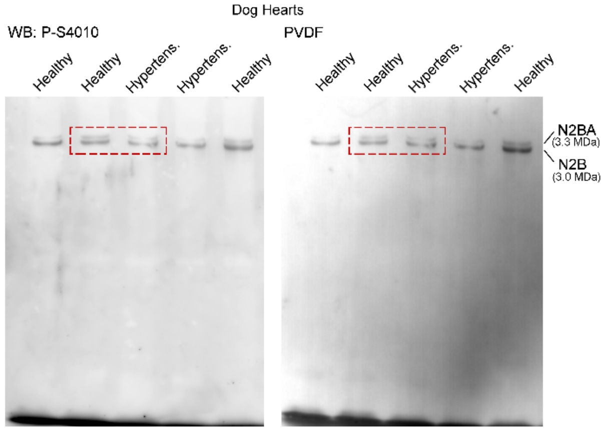

Supplementary Figure 2b

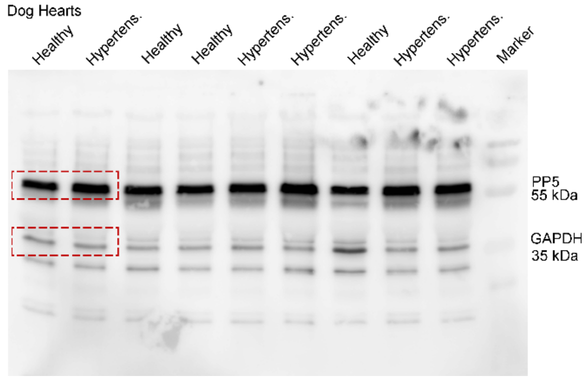

Supplementary Figure 4b

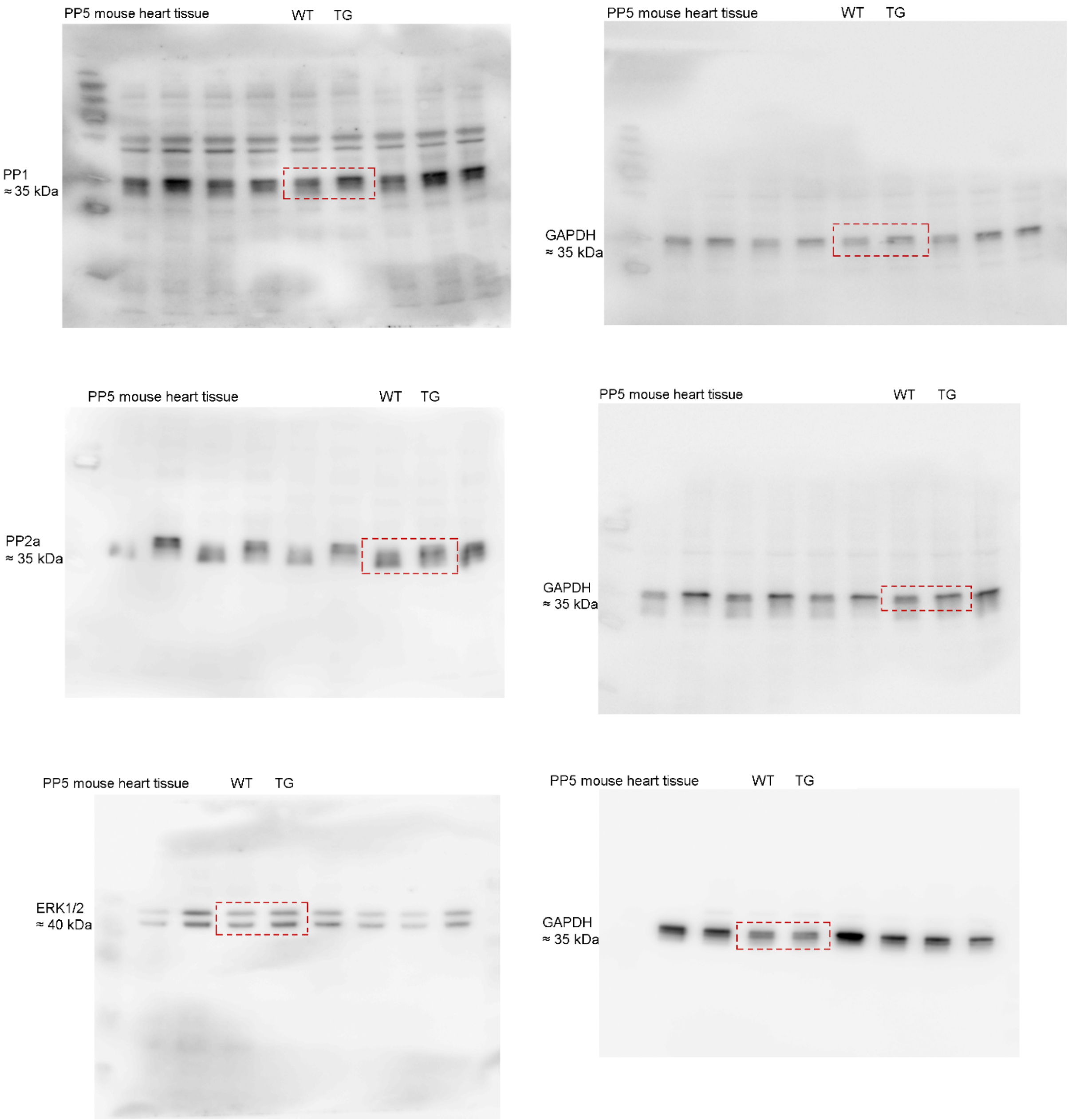

Supplementary Figure 6. Uncropped images of blots and gels used in supplementary figures. The number of the corresponding figure is shown above the panels. Regions used for figures are boxed. Position of molecular mass marker is shown on the side (kDa or MDa).

Supplementary Table 1. Primers for PCR used to generate recombinant WT constructs.

Name	Restriction site	Sequence
Titin N2Bus 5‘<br>Titin N2Bus 3‘	*Nde*I (CATATG)<br>*Sal*I (GTCGAC)	GACATGACTGATACCCCCTGC<br>GCCATCCTCTTTGATTAAGC
Titin N2Bus 5‘<br>Titin N2Bus 3‘	*Bam*HI GGATCC)<br>*Xho*I (CTCGAG)	GACATGACTGATACCCCCTGC<br>GCCATCCTCTTTGATTAAGC
N2Bus C-Term 5‘<br>N2Bus C-Term 3‘	*EcoR*I (GAATTC)<br>*Xho*I (CTCGAG)	CTTGTTACTTCGGCAAAGTCTGTAACAG<br>GCCATCCTCTTTGATTAAGCCACCCTCAG
PP5 full-length 5‘<br>PP5 full-length 3‘	*EcoR*I (GAATTC)<br>*Xho*I (CTCGAG)	ATGGCGATGGCGGA<br>TCACATCATTCCTAGCTGCA
PP5 catalytic 5‘<br>PP5 catalytic 3‘	*EcoR*I (GAATTC)<br>*Xho*I (CTCGAG)	AGCATGACCATTGAGGAT<br>CATCATTCCTAGCTGCAG
PP5 TPR 5‘<br>PP5 TPR 3‘	*EcoR*I (GAATTC)<br>*Xho*I (CTCGAG)	GCAGAGGAGC<br>GTCATGGGGCT
PP5 TPR+ 5‘<br>PP5 TPR+ 3‘	*EcoR*I (GAATTC)<br>*Xho*I (CTCGAG)	ATGGCGATGGCGGA<br>GTCTTCAAGCTTGGGTCC
Hsp90 5‘<br>Hsp90 3‘	*EcoR*I (GAATTC)<br>*Xho*I (CTCGAG)	ATGCCCCCGTGTTCGGGC<br>GTCTACTTCTTCCATG
FHL-2 5‘<br>FHL-2 3‘	*EcoR*I (GAATTC)<br>*Xho*I (CTCGAG)	ATGACTGAGCGCTTTGAC<br>TCAGATGTCTTTCCCACAG
Titin N2A 5‘<br>Titin N2A 3‘	*EcoR*I (GAATTC)<br>*Sal*I (GTCGAC)	GCTGGCAGGGAAATAAAGC<br>GGTGGCACTGCTCTGATAGG
Titin PEVK 5‘<br>Titin PEVK 3‘	*EcoR*I (GAATTC)<br>*Xho*I (CTCGAG)	CCTGGAGGTGAAAAGAAAG<br>GGTGAACGGGGCTT
